# Supplementary material for: Improving average ranking precision in user searches for biomedical research datasets
Source: Database (Oxford). 2017 Nov 6;2017:bax083. doi: 10.1093/database/bax083 (PMC5714153; doi:10.1093/database/bax083)
Supplement: Supplementary Data S1 [file bax083_supp_s1.docx]

Table S1 – Train queries (EA prefix) and test queries (T prefix).

EA1 – Find data of all types related to TGF-β signaling pathway across all databases

EA2 – Find data of all types on synaptic growth and remodeling related to glycolysis in the human brain across all databases

EA3 – Search for data on BRCA gene mutations and the estrogen signaling pathway in women with stage I breast cancer

EA4 – Find data of all types on the regulation of DNA repair related to the estrogen signaling pathway in breast cancer patients across all databases

EA5 – Search for data of all types on multiple sclerosis of all types across all databases

EA6 – Find data on T-cell homeostasis related to multiple sclerosis across all databases

T1 – Find protein sequencing data related to bacterial chemotaxis across all databases

T2 – Search for data of all types related to MIP-2 gene related to biliary atresia across all databases

T3 – Search for all data types related to gene TP53INP1 in relation to p53 activation across all databases

T4 – Find all data types related to inflammation during oxidative stress in human hepatic cells across all databases

T5 – Search for gene expression and genetic deletion data that mention CD69 in memory augmentation studies across all databases

T6 – Search for data of all types related to the LDLR gene related to cardiovascular disease across all databases

T7 – Search for gene expression datasets on photo transduction and regulation of calcium in blind D. melanogaster

T8 – Search for proteomic data related to regulation of calcium in blind D. melanogaster

T9 – Search for data of all types related to the ob gene in obese M. musculus across all databases

T10 – Search for data of all types related to energy metabolism in obese M. musculus

T11 – Search for all data for the HTT gene related to Huntington’s disease across all databases

T12 – Search for data on neural brain tissue in transgenic mice related to Huntington’s disease

T13 – Search for all data on the SNCA gene related to Parkinson’s disease across all databases

T14 – Search for data on nerve cells in the substantia nigra in mice across all databases

T15 – Find data on the NF-κB signaling pathway in MG (Myasthenia gravis) patients
