# Supplementary material for: Improving average ranking precision in user searches for biomedical research datasets
Source: Database (Oxford). 2017 Nov 6;2017:bax083. doi: 10.1093/database/bax083 (PMC5714153; doi:10.1093/database/bax083)
Supplement: Supplementary Data S2 [file bax083_supp_s2.docx]

Table S2 – Optimized parameters for official submission.

| **Model** | **Term frequency normalization (c)** | **Query expansion (k)** | **Classification gain (g)** | **Non key-relevant term expansion loss (l)** | **Key-relevant term weight boost (c)** | **Linear combination coefficient (**α) |
| --- | --- | --- | --- | --- | --- | --- |
| sibtex-1 | 1.00 |  |  |  |  |  |
| sibtex-2 | 0.95 | 10 | 0.10 |  |  |  |
| sibtex-3 | 0.95 | 10 | 0.10 | 0.01 |  |  |
| sibtex-4 |  |  | 0.10 |  | 1 |  |
| sibtex-5 |  |  |  |  |  | 0.5 |
