# Supplementary material for: Improving average ranking precision in user searches for biomedical research datasets
Source: Database (Oxford). 2017 Nov 6;2017:bax083. doi: 10.1093/database/bax083 (PMC5714153; doi:10.1093/database/bax083)
Supplement: Supplementary Data S3 [file bax083_supp_s3.docx]

Table S3 – Optimized parameters for cross-validation experiment.

| **Model** | **Term frequency normalization (c)** | | **Query expansion (k)** | | **Classification gain (g)** | | **Non key-relevant term expansion loss (l)** | | **Key-relevant term weight boost (c)** | | **Linear combination coefficient (**α) | |
| --- | --- | --- | --- | --- | --- | --- | --- | --- | --- | --- | --- | --- |
| sibtex-1 | | 33 | |  | |  | |  | |  | |  |
| sibtex-2 | | 33 | | 25 | | 0.01 | |  | |  | |  |
| sibtex-3 | | 33 | | 25 | | 0.01 | | 0.05 | |  | |  |
| sibtex-4 | |  | |  | | 0.02 | |  | | 1.85 | |  |
| sibtex-5 | |  | |  | |  | |  | |  | | 0.7 |
